# Climate change and allergic diseases: An overview

**DOI:** 10.3389/falgy.2022.964987

**Published:** 2022-10-13

**Authors:** A. B. Singh, Pawan Kumar

**Affiliations:** CSIR-Institute of Genomics and Integrative Biology, Delhi University Campus, Delhi, India

**Keywords:** climate change, noncommunicable diseases, pollen allergy, air Pollution and pollen morphology, respiratory diseases

## Abstract

Climate change has been regarded as a threat to the human species on the earth. Greenhouse gasses are leading to increased temperatures on Earth besides impacting the humanity. These atmospheric conditions have shown to alter the release pattern of pollens and can change the timing and magnitude of pollen release with flowering plants. As pollen is responsible for respiratory allergies in humans, so climate change can adversely affect human health in susceptible individuals. In this review, we highlight the association between climate change, increased prevalence and severity of asthma, and related allergic diseases. Increased air pollution can alter the production of local and regional pollen. This altered pattern depends on bioclimatic parameters. As simulated with a pollen-release model and future bioclimatic data, warmer temperatures lead to an increased pollen count in some specific locations and for longer periods. Thus, anticipation of a future allergic disease burden can help public health agencies in planning to develop strategies in mitigating the unprecedented health challenges expected in future years.

## Introduction

Noncommunicable diseases (NCDs) are of great concern for healthcare professionals worldwide as these are increasing the burden of ill health and poor quality of life. They also contribute to socioeconomic inequality. Limited medical facilities, along with low economic status, are adversely affecting the urban and rural poor in resource-limited countries. NCDs principally include diabetes, cancer, cardiovascular diseases, and chronic respiratory diseases. The rising incidence of respiratory diseases, such as allergic rhinitis and asthma, has been attributed to increasing air pollution, urbanization, and climate change (1). Several epidemiological studies have stressed that global warming, air pollution, and climate change are leading to an increased incidence of respiratory disorders such as rhinitis and asthma, especially among vulnerable groups, including children and older adults (2). Indeed, climate change threatens the gains made in public healthcare in the last 50 years (3).

Allergic diseases have been reported to be caused by genetic susceptibility and environmental factors such as foods, dust mites, pollens, fungi, and animal dander. As these allergenic precursors are important in determining the factors toward the incidence and prevalence of allergic diseases, climate change influences the levels and location of these aeroallergens (4).

The change in climate influences human health in several ways. Primarily, extreme weather events, such as heat to drought, heavy rainfall, flooding, and cyclones, impact natural ecosystems, agricultural productivity, species migration, and the distribution patterns of vector, water-, and food-borne diseases. In addition, climate change may further impact health indirectly leading to undernutrition, mental ill health, health-related unemployment, violence, and conflict (3).

The risk factors for respiratory disorders broadly include occupational agents, indoor pollution from cooking fuel and tobacco smoke, environmental exposure to air pollutants from traffic and fossil fuel burning, and bio-particulates such as aeroallergens (pollen, fungal spores, insects, biological debris, etc.). These are manageable and preventable factors. Unfortunately, steps taken by governmental agencies are not sufficient despite the availability of monitoring the status and health impact reports of such diseases by international bodies such as the World Health Organization (WHO) (5).

As pollens make up an important component of the aeroallergen load in the environment, “pollen grains” (male reproductive parts of plants) as aeroallergens are well studied across the world. Pollens have been attributed to be the main causative agent of pollen allergy or pollinosis. The incidence of pollen allergies has continued rising in recent years due to changing climatic conditions. Pollinosis is the cause of allergic responses, such as rhinitis (hay fever) and asthma, which are considered global health concerns (6–8). In addition, pollens have also been associated with nonallergic respiratory diseases, for example, chronic obstructive pulmonary disease, stroke and myocardial infarction, and even suicide mortality (9, 10).

It is estimated that there are 8 million species of plants and animals on Earth. Unfortunately, the rate of losing a species is 1,000 times faster, a record-breaking figure to date (11). This has been attributed to urbanization, changes in land use, and microclimatic changes induced by human settlement. In addition, air quality is deteriorating as a result of anthropogenic emissions such as industrial pollution, vehicular traffic, fossil fuel for the energy sector, and cooking fuel for domestic use (12). Transport emissions are a major contributor to air pollution and have adverse health implications, such as respiratory and cardiovascular diseases (13). The impact of these climate changes has adversely affected agricultural crop production, diversity, and distribution of natural species, and biological patterns such as flowering time and pollination.

The adverse effects of rising temperatures, elevated levels of carbon dioxide (CO_2_) on phenology with regard to productivity, especially for staple and cash crops, have been assessed (14, 15). However, there are sparse data on the direct correlation between climate change, pollen seasons, and allergic sensitization. This will require years of detailed pollen counts and meteorological monitoring, with simultaneous recording of the clinical data of the resident population of the study site.

The aim of the present article was to highlight the impact of these three major risk factors on pollen production, pollen season, and altered allergenic contents as reported in various studies.

## Prevalence of respiratory disorders

Recent epidemiological studies have pointed toward a general increase in both the incidence and prevalence of respiratory diseases, such as allergic rhinitis (hay fever) and asthma (16–18). Over the last 60 years, there has been a rise in the epidemic prevalence of allergic disorders, which is expected to reach 4 billion in the 2050s (19). Asthma, allergic rhinitis, atopic dermatitis, and inhalant sensitization have been described as the first wave of the epidemic, which will become a pandemic in comparison with infectious diseases in the 21st century (20–23).

## Burden of air pollution on respiratory diseases

The world’s population is exposed to a huge burden of both outdoor and indoor pollutants, including particulate matter with a diameter of 2.5 μm or less (PM2.5), PM10, SPM, CO, O_3_, nitrogen dioxide (NO_2_), SO_2_, NO, and household pollutants such as biomass and tobacco. Air pollution has become a growing concern, especially in urban cities with rapidly developing economies, increasing infrastructure, numbers of vehicles, and reduced green spaces. Nearly half (48%) of estimated O_3_-attributable and over half (56%) of PM2.5-attributable visits to the asthma emergency room were estimated in Southeast Asia (including India) and the western Pacific region (including China).

It is estimated that by the year 2050, 66% of the global population will be living in urban cities, whereby the population and pollution level will be similar (24). The benefits of moving to urban areas or converting a semirural to urban location, offers easy access to healthcare, sanitation, and secure nutrition, while the drawbacks include the hidden effects of a rapidly growing population, rising pollution, social deprivation, criminal offenses, and mental stress-related illness that humans neglect and will affect the quality of life in the long run.

According to the report of the National Commission of Macroeconomics and Health, Government of India, the morbidity rate for chronic respiratory diseases in India is much higher than other noncommunicable diseases, with the number of asthma-affected patients expected to increase from 45 to 65 million by the end of the year 2016 (25). NCDs currently cause more deaths than all other diseases combined and compared with communicable diseases, with a projected increase from 38 million in 2012 to 52 million by 2030. The top three countries in Asia for both the incidence and prevalence of asthma are India, China, and Indonesia, driven largely by population size and rapid urbanization.

## Pollution and climate change

Climate change has resulted in the change of natural ecosystem through greater land degradation, mainly in low-lying coastal areas, river deltas, dry land, and in permafrost geographic areas (26). Increased atmospheric CO_2_, methane, and nitrous oxide concentration, which are the most important contributors of the greenhouses gases, are the largest drivers of climate change by increasing the temperature by uptake of energy by the climate system, which has led to global warming. The average temperature of the Earth's surface is approximately 14 °C and has increased by almost 1 °C over the past 100 years or so. This has resulted in changes in precipitation patterns, either decreasing or increasing precipitation. In addition, these climatic changes have resulted in the increased frequency and intensity of thunderstorms and tropical cyclones (27).

Increased heat, drought, and insect outbreaks, all linked to climate change, have increased wildfires. Recent catastrophic events, such as the extreme changes in sea level and associated devastating events, such as the Australian and California wildfires, hurricanes (Maria, Atlantic), typhoons, and flooding (most recently in Venice, Italy), have raised concerns for human safety and health, especially for more susceptible age groups and distressed socioeconomic classes. The year 2019 has been marked as the hottest and driest year on record, with the Australian bushfires during the Black Summer as one of the most natural catastrophic events with a high probability of recurrence (28). Even highly developed nations with a sound infrastructure will not be spared from the effect of hurricanes (29).

With the aftermath of Hurricane Maria in Puerto Rico in 2017, an increase has been reported in asthma and cases of asthma due to the increased use of diesel- or gasoline-powered generators. The asthmatic consequences ascribed to the weather events of 2018, such as Atlantic hurricanes, volcanic activity in Hawaii, and flooding events in Venice, Italy, are still being interpreted (29).

Asthma epidemics occurring in relation to thunderstorms have been reported from geographical locations such as Europe and Australia, predominantly in seasons when there are high atmospheric loads of airborne pollen, a time unfavorable for those with pollen allergies. The beginning and end of a thunderstorm is critical.

## Effect of climate change and air pollution on respiratory diseases

Air pollution, climate change, and reduced biodiversity arising due to urbanization has led to an increase in the variety of chronic disorders, such as respiratory and cardiovascular diseases (2). Policymakers are carefully working to build strategies to mitigate the issues arising due to climate change (25).

The urban population is continuously exposed to pollutants and reduced biodiversity, which have led to decreased plant, animal, and microbial interaction. This has led to an impaired immune response in the urban population compared with the rural population, as they are exposed to similar levels of pollution but reside in a habitat rich with flora and fauna in comparison with a habitat with reduced biodiversity (30).

A recent position statement on climate change and health impacts from the European Respiratory Society was developed after a workshop co-organized by the HENVINET Project and the American Thoracic Society. The position statement highlights climate-related health impacts, including deaths and acute morbidity due to heatwaves, increased frequency of acute cardio-respiratory events due to higher concentrations of ground-level ozone, changes in the frequency of respiratory diseases due to trans-boundary particle pollution, and altered spatial and temporal distribution of allergens (pollens, molds, and mites) and some infectious disease vectors (31, 32). According to WHO estimates, 3 million people die prematurely every year as a result of air pollution, especially in the major cities of Asia, Africa, and Latin America.

Sulfur oxide and nitrous oxide arising due to the use of fossil fuels are the main sources of air pollution. Increased levels of these are released into the atmosphere, leading to poor health, low air quality, and acid rain. These air pollutants cause direct cellular injury or induce intracellular signaling pathways and transcription factors in sensitive individuals, especially in asthmatic patients.

A global study stated that a staggering 9–33 million (8%–20%) of total global emergency asthma visits were attributed to O_3_ alone and 5–10 million (4%–9%) of total global visits to the asthma emergency room were associated with PM2.5 in 2015. Epidemiological and clinical experimental studies have revealed that countries in Southeast Asia, including India and China, contribute toward approximately half (48%) of estimated O_3_-attributable asthma emergency visits and slightly more than half (56%) of PM2.5-attributable asthma emergency room visits. Furthermore, 23% and 10% of global asthma emergency room visits were estimated to be associated with O_3_, 30% and 12% for PM2.5, and 15% and 17% for NO_2_ in India and China, respectively (33). The incidence of pediatric asthma attributable to PM2.5 levels was estimated to be approximately 57% in India, 51% in China, and more than 70% in Bangladesh. Asthma, acute respiratory tract infections, chronic obstructive pulmonary disease, and the exacerbations of preexisting obstructive airway disease and lung cancer are adverse respiratory effects of air pollution. PM2.5 has been associated with premature morbidity (2, 34).

It is evident that the changes in climate and air quality have drastic and quantifiable impacts on the incidence of morbidity and respiratory diseases (32).

## Effect of climate change pattern on plant morphology with special reference to pollen, pollinosis, and allergenic sensitization

The bio-particulates that cause allergic symptoms are pollens, fungal spores, insect debris, house dust mites, animal dander, and foods (8). Pollen grains have been found to cause asthma, and allergic rhinitis and allergic conjunctivitis in atopic patients (35). The distribution and prevalence of pollen allergies are subject to both geographical and chronological variations (36–38).

Aeroallergenic particles, such as pollen, pollen-derived submicronic (<10 μm) and paucimicronic (<1 μm) particles, can reach the lower airways, eliciting allergic symptoms in susceptible individuals. These particles are mainly composed of starch granules and polysaccharide particles, which may be absent in mature pollen (34, 39).

Allergenic molecules released from pollen are responsible for the sensitization and elicitation of allergic symptoms in humans. The release of allergens from pollens has been thought to occur either primarily outside the individual organism when pollen grains are spreading through the atmosphere or, second, when pollens come into contact with the mucosal surface of the upper respiratory tract.

Pollen grains generally belong to the coarse fraction of air particulate matter (particle diameters >10 μm), but fungal spores and pollen fragments are also found in fine particulate matter (<2.5 μm; PM2.5), which can penetrate deep into the human respiratory tract and alveolar regions of the lung (40). The wall of pollen is divided into two layers: the inner intine layer and the outer sculptured exine layer. The inner intine layer is rich in cellulose while the outer wall is composed of sporopollenin, a highly tough and resistant biopolymer (41–43). Intine is composed of cellulose and lignin. Sporopollenin is a polymerization product of carotenoid hydrocarbons and carotenoid esters.

The presence of allergenic proteins in pollen and the variation in the levels of expression of these molecules may depend on plant species, the maturation stage of flowers, environmental and climatic factors, and pollution. Information about change in climatic conditions, daily variations, and seasonal prevalence becomes very important in handling patients with pollinosis (44).

Elevated concentrations of atmospheric CO_2_, which are a result of the use of fossil fuels, may boost plant growth and in turn lead to increased pollen production, which may affect anther dehiscence patterns. This may affect pollen dispersal and transport and increase the duration of the pollen season. They may also lead to the emergence of new pollen species in new locations that may not be endemic to the specific area (2, 34). As reported, the onset, duration, intensity of pollination, and flowering patterns, along with the sporulation of fungi, may alter the allergen content and allergenicity of pollen grains, fungal spores, and other bio-particulate matter as a result of changing climatic conditions (30, 45, 46).

A long-term, detailed, cause-effect study spanning 27 years (1981–2007) was carried out to assess the effect of climate variables on pollen counts, meteorological factors, and allergic sensitization rate in western Liguaria (northwest Italy). The study revealed that climatic variables may increase the duration of pollen seasons for *Parietaria* by 85 days, olive (18 days), and cypress (18 days). They reported constantly increased pollen sensitization throughout the year, with an increasing pollen load in comparison with stable sensitization to the house dust mite (47).

In Europe, the main pollination period spans half the year, from spring to autumn. The distribution of allergenically important airborne pollen taxa has been studied across six geographical locations: the Arctic, Central Europe, Eastern Europe, the mountains, and the Mediterranean areas (44). Olive has economic importance to the Mediterranean region. However, olive pollen has been associated with allergenic properties and possesses greater risks to human health (44). A study carried out from 1999 to 2008 across 16 olive-producing cities of Italy presented updated information on *Olea europaea* pollen production and pollinosis along with the effect of climate characteristics. The study reported and analyzed the pollen count, the start and end date of the flowering season, with the maximum daily pollen concentration meaning full flowering (FF). The pollen season for *Olea* ranges from late April to mid-June, with the last 10 days of May posing greater health risks. An upscaling of the pollen season in the atmosphere under the influence of climatic parameters such as temperature and latitude has been observed. The allergy season for these types of plants with a 2-year reproductive cycle might be significantly precocious in future decades (20–30 days earlier in the year), which will impact on the severity and duration of allergies attributable to olive tree pollen. In the different areas studied, an increase in temperature by 1 °C on average during the pollen-release months (March, April, and May) produced precocious FF dates that were 5.5–7.6 days earlier in the year.

The airspora in Southeast Asia, a tropical region, has not been well reported. Trees and shrubs flourish year-round with an abundance of ferns (48–50). There is no distinct definable major flowering season for plants (48, 51) in comparison with temperate countries.

An investigational study from Gyeonggi Province, Seoul, Korea, from 1999 to 2008 revealed a possible correlation of skin test positivity results and hospital visits of tree pollen allergy patients as a result of temporary meteorological variations. This study revealed that the increased minimum temperature in the preflowering period may be associated with elevated pollen counts, which may be leading to increased tree pollen sensitization and hospital visits (52).

An interesting observation regarding the effect of climate on pollen counts from small Jeju Island, Korea, revealed that the southern region showed a slightly longer period with a higher pollen count of Japanese cedar (JC) pollen compared with the northern region in a study carried out from 2011 to 2013. A comparative analysis from Jeju city in the northern region (NR) and Seogwipo city in the southern region (SR), where the mean temperature during efflorescence season for JC pollen varies by approximately 2.0 °C, showed a significantly higher aeroallergen sensitization toward JC pollen in the SR compared with the NR. This has been attributed to slightly different climatic conditions in different regions. In addition, JC pollen is considered the most frequent sensitizer outdoor aeroallergen in Jeju city with sensitization rates as high as 33.8% in Jeju (a temperate geographical island) compared with only 1.1% in Seoul and 0.7% in Suwon.

Another study from 2008 to 2013 from Seoul, Korea evaluated the changes in pollen count and skin prick test (SPT) patterns. A correlation was observed among the annual increase in pollen count for trees, grasses, and weeds and skin positivity rates. Increased SPT positivity may be either due to increased pollen allergenicity or efficient pollen dispersal and longer suspension in the air due to climate change (34, 36).

A recent study from Japan showed that seasonal allergic rhinitis, which is caused by JC pollen (i.e., sugi-pollinosis) is alarmingly affecting one-third of all Japanese individuals and it has been increasing for the past 20 years. Sugi-pollinosis has been attributed to an increased number of cedar pollens as a result of global climate change and tree-planting programs initiated by the Japanese government after World War II (53).

Therefore, it is hypothesized that global climate change may influence human health by increasing the average temperature. This will lead to earlier flowering, increased pollen scattering in the air, and a longer efflorescence season. Further climate change may lead to increased CO_2_ emissions, directly inducing photosynthesis and plant growth (50, 54).

## Climate change and pollutants affecting the allergenicity of plants

In order to monitor the occurrence and abundance of pollens in the ambient air for the early warning of allergic diseases as well as other health risks, a limited number of studies have been conducted. A planned routine study of the diurnal, seasonal, and annual fluctuations in the concentration of atmospheric pollen, especially allergenic plants, in relation to meteorological factors will always be a governing factor in the correct diagnosis and therapeutic management of patients experiencing pollen-induced respiratory allergic disorders (55).

With a continuously changing climate, such information is essential for the timely prevention of allergic disorders, with a better patient outcome in this era of customized precision treatment. Classical relevant studies in this direction are reviewed and highlighted further in the section. Data provided from 30 years of observations within the International Phenological Gardens Network showed that spring events advanced by 6 days, with the highest rate of phenological changes being observed in Western Europe and the Baltic region. Conversely, phenological trends appear to be different in the eastern border of Europe, sometimes showing a start of the phases 1–2 weeks later. Due to its earlier onset, the pollen season is more often interrupted by adverse weather conditions in late winter/early spring (45). The duration of the pollen season is also extended, especially in summer and in late-flowering species.

The much-gained attention from the European and Mediterranean Plant Protection Organization is for the alien, invasive, and noxious plant species *Ambrosia artemisiifolia* L. (common ragweed) with a highly allergenic pollen (18). Ragweed, a native of North America, has been invading large areas of South America and Europe for the last few decades and has been identified as a major contributor to severe respiratory allergy diseases. The plant has been rightly described as a successful dominant species in abandoned lands even under severe ecophysiological conditions of an extreme and unpredictable environment (34, 56).

The species has naturalized across Europe at a rapid rate, and accounts for up to 80% of sensitization rates. The most important allergen is Amb a1. In another German Health Study from DEGS, IgE sensitization rates to *Ambrosia artimisiifolia* were 8.2% among German adults, with the prevalence rising unhindered even at very low concentrations (5–10 pollen grains per m^3^ of air), sufficient enough to trigger allergic reactions in sensitive patients. Sikoparija et al. reported an increased prevalence and incidence of asthma with this new allergen at a high frequency compared with other pollen types (57). Furthermore, the effect of doubling CO_2_ levels led to a significant increase in the pollen count, while another study reported with increasing CO_2_ an increase in major allergen concentration, Amb a1, with no change in the total protein level (58). Furthermore, ragweed pollen collected along high-traffic roads showed a higher allergenicity than pollen sampled in vegetated areas; this is probably due to traffic-related pollution. The overall impact will be an altered pollen season timing and load, and hence change in exposure (30).

Further along with allergenic proteins, pollen and fungal spores also contain other compounds that can act as adjuvants. The release of these nonallergenic but bioactive, pollen-associated lipid mediators has proinflammatory and immune modulatory effects that can trigger and enhance allergies. The release of these substances has been shown to be influenced by air pollution with significantly higher levels present in the pollen collected from areas of heavy traffic.

## Effect of climate change, pollution on flowering behavior, and allergic sensitization in India

Several survey-based studies have been reported on hospital admissions and associated pollen season and dominating allergenic plants from different cities of India. Agriculture biotechnologists have been focusing on the development of novel varieties tolerant to abiotic stress factors such as extreme temperature and adverse climate events. Sporadic studies have analyzed climate change-induced changes in pollen morphology and exacerbation in respiratory symptoms in the Indian population/context.

*Populus deltoides* is a fast-growing woody species of tropics in northern India possessing commercial importance. Its flowering time is in early spring, before the appearance of foliage, while buds appear near axil or developing shoots. In a planned observational study during October 2019, an unusual flowering behavior was observed in a plantation trial of *P. deltoides* located at a farmland in the Kapurthala region of Punjab, north India. The male catkins were significantly longer and wider than usual, symbolizing flowering and the ready release of pollen grains in October. The authors suggested the variation in climatic factors, such as the high/low temperature and precipitation duration, forced this off-season behavior by affecting the phytohormone concentration of gibberellic and salicylic acids (59).

A recent study in Chandigarh, an urban Indian city, evaluated the effect of meteorological parameters and air pollutants on airborne pollen. They observed a statistically significant positive correlation with temperature for Cannabis sativa, *Parthenium hysterophorus*, *Poaceae*, and total pollen concentration. Interestingly, NOx levels showed a positive correlation with the total pollen counts for *Celtis occidentalis* only, while major taxa correlated negatively with PM10 and PM2.5 and the total pollen concentration except for *Eucalyptus* sp. A study understanding the effect of air pollution on the aeroallergenic plant *Cassia* growing abundantly in an experimental industrial area and control residential area of Nagpur, Maharashtra, India, reported on the change in pollen morphology. Based on light microscopy and scanning electron microscopy (SEM), two species of *Cassia*, viz., *Cassia siamia* L. and *Cassia fistula* planted in the control area, showed a complete trizonocolporate exine condition of the pollen, whereas in the experimental location a reduced pollen size with breakage of exine was observed. SEM studies also revealed the attachment of some particulate matter to the pollen surface (60).

In another study involving animal experiments and the strong effects of air pollution on the pollen proteins of the Cassia plant, in Pune, India reported increased pollinosis. Serological parameters, such as total white blood cell and lymphocyte count, IgE antibodies, and mast cell degranulation leading to heightened hypersensitivity, were higher in animals that received a pollen-based protein extract from a polluted site compared with a nonpolluted site (61).

*Cassia sophera* is the most widely distributed and common variety growing in the tropical region (62) and can be used as an indicator species because of its relative abundance and the effect imposed on it by the reduction in viable pollen formation (63–69). Moreover, this herb has been selected for its wide use, ready availability under controlled and environmentally stressed conditions, and its height being below the exhaust pipe of vehicles.

The aim of the biomonitoring study was to detect morphological deformities in the leaf and pollen micromorphology of *Cassia sophera* Linn. after exposure to vehicular air pollution. The study areas were selected based on three different roadsides: a control area designated “C” at Serampore College campus as a low vehicular movement area; a bi-lane roadside 1.5 km away from the Durgapur Expressway as a moderate vehicular movement designated “M”; and a roadside near the Durgapur Expressway designated “H” because of continuous vehicular movements. These studies attempted on *Cassia sophera* exposed to different levels of roadside air pollution were used to detect varying degrees of damage produced in its morphological characters with special reference to leaf shape and visible injuries as well as micromorphological features of pollens at wet and dry conditions. In the results, the damage observed was noticeable, with chlorotic spots, necrosis, etc. It not only showed the morphological damage in the leaf but also huge differences in the production of viable pollens with plants exposed to high levels of air pollution showed pollen production with no material content inside as well as with significantly (*P* < 0.001 and *P* < 0.01) reduced sizes compared to the plants exposed to low levels of air pollution.

The effect of air pollution was assessed *in vitro* by Bist et al. (70) on the pollens of *Ricinus communis*. They observed a significant decrease in the soluble protein content with an increase in the concentration and time of exposure in the pollen exposed to SO_2_ and NO_2_. It may be hypothesized that proteins lost from exposed pollens may be deposited on ultrafine respirable dust particles thus increasing allergenicity (70, 71).

It is concluded that varying degrees of air pollution produce a huge impact at the subcellular, cellular, and tissue levels.

## Future for predicting abundances of allergenic pollens: ragweed’s “top-down” approach

*Ambrosia artemisiifolia* is a widely distributed weed and responsible for highly allergenic pollen grains; it affects human health and agriculture in affected areas. The accurate mapping of ragweed densities may help in efficient mitigation measures. A recent algorithm-based prediction has achieved significant success in mapping ragweed quantities with superior resolutions up to 1 and 10 km. These “top-down” approaches integrate pollen data from 349 stations in Europe, which include information regarding habitat and landscape, land cover data, and expert knowledge. This collective data monitoring revealed that northern and southern European countries show the lowest ragweed quantities while Russia, parts of Ukraine, and the Pannonian Plain show the highest. In addition, smaller hotspots are found in the Rhône Valley, Turkey, and northern Italy and France. This kind of predictive modeling can help government agencies develop proper planning and mitigation of health emergencies. This top-down approach is also applicable to other anemophilous species (72, 73).

Another predictive modeling using MAXENT software based on bioclimatic variable minimum temperature, growing season, and water balance-based species distribution models predicted the abundance of three ragweed species. This model predicts that by the year 2100, the distribution of three ragweed species will increase toward northern and eastern Europe under all climate scenarios and Europe will be affected by severe ragweed-associated allergy problems, affecting millions of people (58, 74).

## Conclusion

Climate change has drawn the attention of biologists, environmental activists, and political leaders across the globe. Environmental pollution and increased temperatures have contributed immensely to the climate change and thus have impacted human health significantly. These environmental changes can cause an increase in the production of pollens and change the molecular characteristics of pollen that may increase their allergenic properties. This has been attributed to increased and faster plant growth, an increase in the amount of pollen produced by plants, earlier and longer pollen seasons, and an increase in the amount of allergenic proteins contained in pollen.

Recent predictive modeling based on bioclimatic variables has revealed that climate change will further lead to an increased incidence of allergy as a result of increased pollen counts of some of the important pollens such as ragweed in specific areas of Europe by end of this century.

Thus, there is an urgent need to make the population aware of the effects of climate change and emerging threats. Patients with pollen allergies should be educated about the risk of asthma exacerbation and especially should be warned of the danger of staying outdoors without the proper treatment for their chronic rhinitis and asthma during pollen season.

In addition, government agencies responsible for implanting policies should plan adaptation and mitigation measures. However, these measures may not be able to eliminate all negative impacts, but mitigation measures are crucial to limit changes in the climate system.
